# Correlation analysis of electronic health literacy, compliance behavior, and quality of life in middle-aged and older patients with coronary heart disease: a cross-sectional study

**DOI:** 10.3389/fpubh.2025.1680950

**Published:** 2025-12-08

**Authors:** Suihua Sun, Ruru Guo, Yinan Wang, Leilei Jiao, Chenchen Zhao, Shuxin Qiao, Jingjing Wang

**Affiliations:** 1 Department for Teaching and Research in Community Nursing, School of Nursing and Health, Zhengzhou University, Zhengzhou, China; 2 Catheterization Laboratory, Department of Cardiology, Nanyang Central Hospital, Nanyang, China; 3 Department of Radiation and Medical Oncology Ward, The First Affiliated Hospital of Ningbo University, Ningbo, China

**Keywords:** CHD, EHL, middle-aged and older adults, compliance behavior, QOL

## Abstract

**Introduction:**

The health status and quality of life (QOL) of middle-aged and older patients with coronary heart disease (CHD) are significantly influenced by adherence to medical advice. In the digital age, electronic health literacy (eHL) is increasingly recognized as a crucial factor affecting compliance behavior and QOL; however, research on the relationship between eHL, compliance behavior, and QOL in CHD patients is limited. This study aimed to explore these correlations among middle-aged and older CHD patients and identify strategies to enhance eHL for better health outcomes.

**Methods:**

A cross-sectional study was conducted from May to September 2024 at two tertiary hospitals in Nanyang and Ningbo, China. Participants completed the eHEALS, Morisky Medication Adherence Scale-8, a non-medication adherence questionnaire for secondary prevention in CHD, and the Seattle Angina Questionnaire. Categorical variables are presented as frequencies and percentages, and continuous variables are expressed as medians with interquartile ranges (IQR); Correlations were analysed via Spearman tests; regression models assessed the impact of demographic factors.

**Results:**

Among the 594 participants, 341 were male and 253 females. The median eHL score was 21; medication and non-medication compliance scores were 5.5 and 8, respectively; average QOL scores were 47.73. eHL was significantly and positively correlated with medication compliance (*r* = 0.397**, *p* < 0.001), non-medication compliance (*r* = 0.337**, *p* < 0.001), and QOL (*r* = 0.539**, *p* < 0.001).

**Discussion:**

Middle-aged and older CHD patients show low eHL, poor adherence, and moderate QOL. The findings of this study indicate the positive correlation between eHL, compliance behavior and QOL in middle-aged and older adults with CHD. These findings indicate that enhancing eHL can improve self-management and treatment adherence, thereby enhancing their overall QOL. Therefore, we recommend that medical institutions implement tailored intervention strategies for different patient groups to provide personalized health guidance aimed at comprehensively managing risk factors and promoting physical well-being.

## 1 Background

Coronary heart disease (CHD) is a condition that significantly impacts an individual’s physical and mental health, as well as their overall QOL. CHD is a group of heart diseases primarily caused by coronary atherosclerosis, leading to vascular stenosis or blockage, which results in myocardial ischaemia, hypoxia, and potential necrosis. In recent years, advancements in medical technology have contributed to a reduction in the mortality rate associated with CHD. Nevertheless, CHD continues to pose a substantial global challenge. Annually, a large portion of this burden falls in low- and middle-income countries, accounting for nearly 7 million deaths and 129 million disability-adjusted life years (DALYs) annually (1), with the resulting fatalities, loss of productivity, and economic repercussions far exceeding those associated with other diseases (2). Many middle-aged and older patients face psychological distress, such as anxiety and depression (3), due to prolonged treatment and frequent medical check-ups. This distress significantly impacts their overall quality of life (QOL). Patients with CHD require access to health information to maintain and improve their health status, which places considerable demands on their health literacy (HL) (4). HL refers to an individual’s capacity to acquire, comprehend, analyse, and utilize healthcare information to make informed health decisions (5). In the 21st century, a growing number of individuals are seeking health information online to manage their own health (6). According to statistics, over 354 million Chinese Internet users were aged 50 and above, accounting for 32.5% of the total Internet users in China (7). Although older adults are able to access online health information, they encounter several barriers, including low levels of trust, unfamiliarity with the Internet, and limited HL (6). These factors increase their susceptibility to the influence of ‘pseudohealth information’, which may lead to unhealthy behaviors. In general, pseudohealth information is interpreted as false health information without a factual basis; however, in the real world, much pseudohealth information is fabricated based on certain facts, which only stretches, distorts, exaggerates, and even fabricates the facts (8). To maximize the potential of the Internet, the Chinese government has created “Internet Plus” designs to transform, modernize, and equip traditional industries (9). In the era characterized by “Internet Plus,” electronic HL (eHL) has garnered increasing attention. eHL is defined as an individual’s ability to obtain, understand, evaluate, and apply health information within a digital context (10). A study (11) indicated that in low-income countries, patients with chronic diseases tend to utilize the Internet less frequently and exhibit lower eHL. Addressing the skill gap in eHL among patients with CHD could facilitate their ability to locate and assess relevant online resources to make decisions that are conducive to their health.

In the information age, guiding middle-aged and older adults patients with coronary heart disease to comply with medical advice and access efficient health services can help delay disease progression. Compliance behavior refers to the extent to which patients adhere to the prescriptions and medical orders provided by their physicians during the disease prevention and treatment process (12). This encompasses various aspects, including timely medication intake, maintaining a balanced diet, engaging in moderate exercise, and attending regular follow-up examinations. Research indicates that patients with CHD who consistently adhere to their prescribed medication regimens experience a substantial decrease in the incidence of cardiovascular events (13). Furthermore, it has been observed that adherence to medical advice tends to decline with advancing age (14, 15). Older adults individuals may experience cognitive decline, such as memory impairment or reduced comprehension skills, which can result in forgetfulness or misinterpretation of medical guidance, thereby adversely affecting adherence and QOL.

QOL refers to the influence of individuals’ perceived health conditions or symptoms on their daily activities, encompassing experiences and feelings across various dimensions, such as physiological, psychological, and social aspects (16). Patients with CHD frequently present with multiple comorbidities, including diabetes and hypertension. Compared to other medical conditions, the QOL of patients with CHD is relatively poor. QOL serves as a critical indicator for assessing the health status of middle-aged and older adults diagnosed with CHD (17). Adherence to prescribed medications, dietary modifications, and active engagement in exercise are recognized strategies aimed at enhancing the QOL of these patients (13). However, research has indicated that non-compliance with treatment regimens is relatively prevalent among those affected by CHD (18, 19). Only 43% of the patients adhered to the treatment (19). A significant number of patients may struggle to read or comprehend prescription instructions effectively; consequently, they are susceptible to issues such as improper medication use and poor adherence rates (20). Low compliance levels have been linked to increased rates of hospital readmission and increased mortality associated with myocardial infarction (21, 22). Research has demonstrated negative correlations between eHL and cardiovascular risk, cardiac events, anxiety, and depression (23). Greater eHL is associated with more effective information acquisition, increased learning capabilities, and superior information processing skills. Patients with greater eHL are better equipped to discern the information provided by healthcare professionals, develop a deeper understanding of their condition, recognize the importance of adhering to medical advice, and consequently, follow such guidance more effectively. This promotes the adoption of healthy behaviors.

At present, there are significant disparities in eHL levels among individuals (24), which may influence their adherence to medical recommendations and overall QOL. Studies have demonstrated that advanced age and lower levels of educational attainment are associated with diminished electronic health literacy (eHL) (25). eHL is linked to patients’ online information-seeking behaviors, the doctor-patient relationship, patient adherence to treatment regimens, and overall health outcomes (26). Notably, female patients and non-smokers exhibit significantly higher rates of medication compliance (27), and ageing and divorce negatively impact the quality of life (28). In our analysis, we included these factors as potential influencing variables to elucidate their relationships with eHL, compliance, and quality of life among middle-aged and older adults patients diagnosed with coronary heart disease. Previous research on eHL has predominantly focused on defining its concept and assessing it within the general population (29, 30). Moreover, studies examining compliance behaviors have largely focused on isolated actions such as timely medication adherence; however, there remains a notable gap in understanding the factors influencing QOL, particularly concerning correlations with eHL and compliance behavior. First, most previous studies examined the relationships between eHL, adherence, and QOL separately, without integrating these variables into a unified analytical framework. Second, research on middle-aged and older patients with CHD remains scarce, although this population often experiences both digital literacy challenges and complex treatment regimens. Third, most available evidence has been derived from Western populations, and there is limited understanding of how eHL affects adherence and QOL within Chinese cultural and healthcare contexts. Therefore, the present study aimed to fill these gaps by comprehensively investigating the associations among eHL, medication, non-pharmacological adherence, and QOL in middle-aged and older adults with CHD. Based on prior findings, we propose the following hypotheses: H1, Higher eHL is positively associated with better medication adherence; H2, Higher eHL is positively associated with better non-pharmacological adherence; H3, Higher eHL and higher adherence are associated with improved QOL. This study clarifies the relationships between eHL, adherence, and QOL, offering practical insights for targeted digital health interventions to enhance the well-being of patients with CHD.

## 2 Methods

### 2.1 Participants

**Figure 1:**
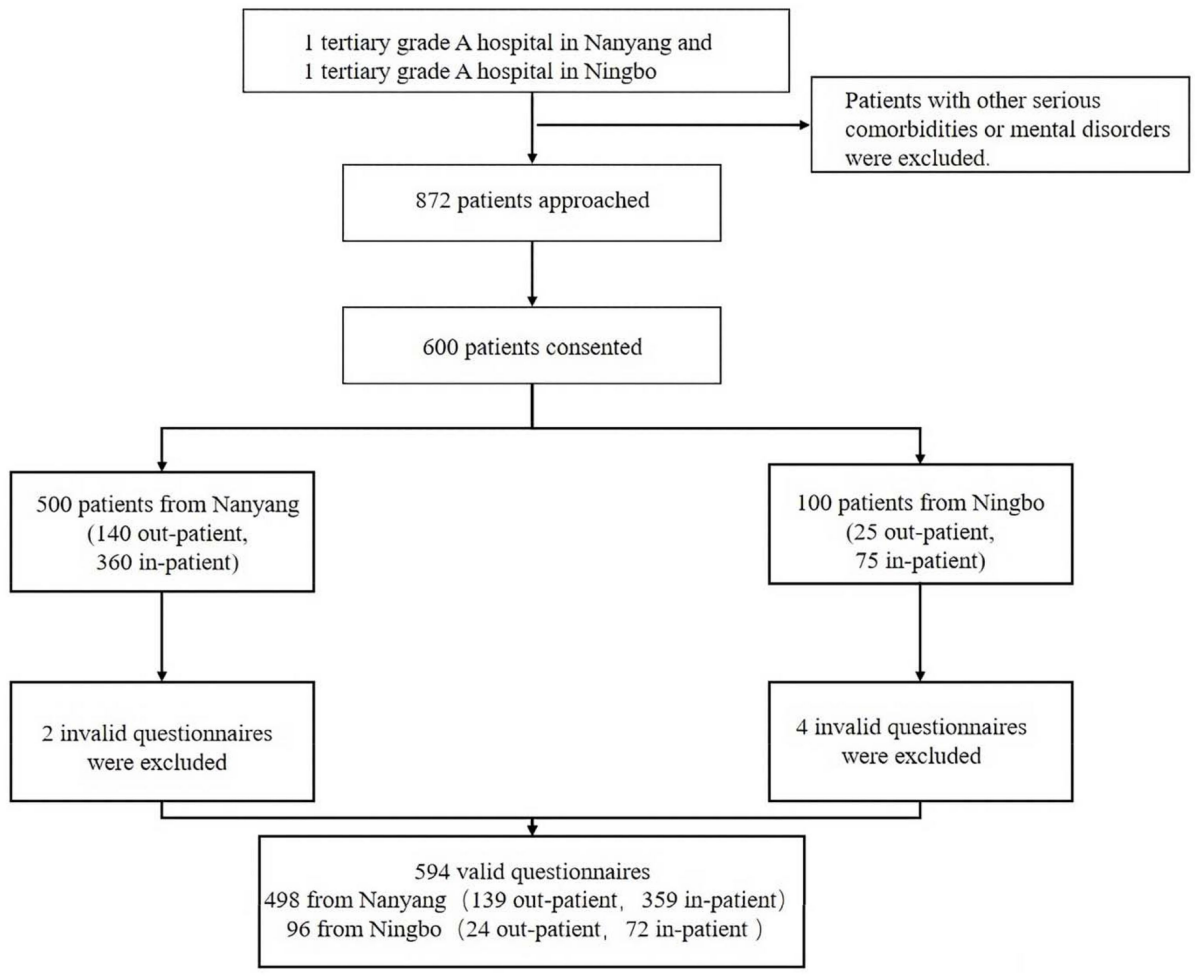
Flowchart for recruiting survey participants.

This study recruited middle-aged and older patients with CHD from two tertiary Grade A hospitals located in Nanyang and Ningbo, China. In the Chinese healthcare system, tertiary Grade A hospitals represent the highest level of general hospitals and serve as regional centres that provide comprehensive care for large numbers of chronic disease patients. Health information is mainly delivered through physician-patient communication and official hospital or government platforms, which differs from Western settings, where patients often obtain health information independently. From May to September 2024. The inclusion criteria for this study were individuals aged ≥ 45 years who had been diagnosed with CHD and possessed adequate comprehension and communication skills. Conversely, patients with other complications or coexisting mental disorders were excluded from the study. The recruitment of participants included inpatients and outpatients from tertiary grade A hospitals in two cities, using consistent inclusion and exclusion criteria. A total of 600 patients were recruited: 163 outpatients and 431 inpatients, with an average hospital stay of approximately 6 days (Figure 1).

### 2.2 Sample size

The sample size estimation in this study was based on the main research hypothesis that a higher level of eHL is associated with better health-related QOL in patients with CHD. Logistic regression was used as the primary analytical model, with QOL as the dependent variable and eHL as the key independent variable. According to prior evidence on eHL and health outcomes, an OR of approximately 1.5 was assumed for the association between eHL and higher QOL, with an expected baseline proportion of higher QOL of about 0.40. Using Hsieh’s method for logistic regression (two-sided *α* = 0.05, power = 0.80) and including about ten covariates (e.g., age, sex, education, income, smoking, drinking, marital status, region, CHD awareness, and Internet information seeking), the minimum required sample size was estimated to be approximately 250–300 participants. Allowing for a potential 15% rate of invalid questionnaires, the target sample size was set at ≥ 345. Finally, 594 valid questionnaires were collected, exceeding the required number and providing sufficient events per variable (EPV ≈ 24 > 10) to ensure adequate statistical power and model stability.

### 2.3 Study design

This cross-sectional study was conducted using a convenience sampling method to administer a questionnaire survey among hospitalized middle-aged and older patients diagnosed with CHD. The questionnaire was completed anonymously by the respondents themselves. Prior to its completion, the researchers presented the principle of confidentiality and the research purpose to the participants and obtained their signatures on the informed consent form. This study was approved by the Ethics Committee of Zhengzhou University (ZZUIRB 2023–306). This cross-sectional study was designed and reported in accordance with the Strengthening the Reporting of Observational Studies in Epidemiology (STROBE) (31) guidelines.

### 2.4 Instruments

#### 2.4.1 Socio-demographic characteristics

The demographic characteristic questionnaire encompasses various factors, including sex, age, marital status, educational attainment, occupation, place of residence, family income, payment method, family history of CHD, smoking and drinking habits, understanding of CHD, frequency of medical visits, duration since diagnosis of the disease, treatment experiences related to CHD, and whether health information is sought online.

#### 2.4.2 eHealth Literacy Scale (eHEALS)

The eHealth Literacy Scale (eHEALS) was developed by Norman and Skinner (32) to measure patients’ eHL abilities in terms of application, evaluation, and decision-making. As a widely recognized instrument for assessing eHL, the eHEALS has been translated into multiple languages and extensively utilized in research contexts (33, 34). In this study, we adopted the validated Chinese version of the eHEALS, which has been widely applied among Chinese populations and has demonstrated good reliability and validity. A 5-point Likert scale was used for scoring purposes. According to previous studies (25, 35), a score of ≥ 26 indicates adequate eHL, whereas a score of < 26 signifies inadequate proficiency. The Cronbach’s *α* coefficient for this scale was reported to be 0.913.

#### 2.4.3 Morisky Medication Adherence Scale-8 (MMAS-8)

The MMAS-8 was developed by Morisky et al. (36) and has been extensively utilized in research related to various chronic diseases (37, 38). The MMAS-8 consists of eight items. Each of the first seven items had two possible responses (yes/no), while the eighth item was answered using a five-point Likert scale. The total medication adherence score ranged from 0 to 8, with higher scores indicating better adherence. A total score of < 6 is considered low adherence, a score between ≥ 6 and < 8 represents moderate adherence, and a score of 8 indicates high adherence (36). The Cronbach’s *α* coefficient for this scale was 0.83.

#### 2.4.4 Non-medication adherence questionnaire for secondary prevention in CHD

A questionnaire on non-medicine adherence in the secondary prevention of coronary heart disease (39) was used to evaluate patients’ non-pharmacological adherence across five dimensions: smoking cessation and alcohol limitation, physical exercise, a balanced diet, emotional regulation, and regular follow-up appointments. This scale was developed by Yang (39). A score of < 9 was classified as low compliance, scores between ≥ 9 and < 12 were deemed moderate compliance, and scores ≥ 12 were categorized as high compliance. The Cronbach’s *α* coefficient for this scale was 0.802.

#### 2.4.5 Seattle Angina Questionnaire (SAQ)

The Seattle Angina Questionnaire (SAQ) (40) was recognized as a specific instrument for assessing QOL in patients with CHD. It evaluates this quality across five dimensions: the extent of physical activity limitation (PL), the stability of angina pectoris (AS), the frequency of angina pectoris episodes (AF), the level of treatment satisfaction (TS), and patient awareness regarding their condition (DP). Each item was assessed using an ordinal value ranging from 1 to 5 or 6, with higher item scores representing a high level of function or satisfaction. Five subscale scores were calculated separately, and no total scores were generated: first, summing item scores within each subscale; second, transforming subscale scores to 0–100 by subtracting the possible lowest values, dividing by the range of the subscale, and multiplying by 100 (40). The scoring system includes several categories: very poor to poor (0–24), fail (25–49), good (50–74), and excellent (75–100). In the present study, we used the Chinese version of the scale translated by Rao and Yuan (41). Liu (42) conducted reliability and validity tests on this scale, which demonstrated a Cronbach’s *α* value of 0.759.

### 2.5 Data analysis

All data were double-entered independently using EpiData 3.1 and analysed with IBM Statistical Package for the Social Sciences (SPSS), version 27.0 (IBM Corp., Armonk, NY, USA). Categorical variables are presented as frequencies and percentages, and continuous variables are expressed as medians with interquartile ranges (IQR). The normality of continuous variables was evaluated using the Kolmogorov–Smirnov test. Because several variables (including eHL, medication adherence, non-medication adherence, and QOL) were not normally distributed, non-parametric tests were used for group comparisons. The Mann–Whitney U test was used for two-group comparisons (e.g., male vs. female), and the Kruskal–Wallis H test for comparisons among three or more groups (e.g., age or education levels).

Spearman’s rank correlation was used to examine the associations among eHL, medication adherence, non-medication adherence, and QOL. Binary logistic regression was conducted to identify factors associated with adequate eHL (≥ 26 vs. < 26 points). Ordinal logistic regression models were applied to assess the effects of demographic and behavioral characteristics on medication adherence, non-medication adherence, and QOL, which were treated as ordered categorical outcomes (low, moderate, and high). The proportional odds assumption was tested before model fitting, and multicollinearity was assessed using variance inflation factors (VIF < 5). Model fit was evaluated using the Hosmer-Lemeshow test and Nagelkerke *R*^2^. A two-tailed *p* < 0.05 was considered statistically significant. All statistical analyses were conducted under the supervision of a professional biostatistician to ensure methodological rigour and accuracy of the results.

## 3 Results

Ultimately, 600 patients participated in this study. Six incomplete questionnaires were discarded, resulting in the retrieval of 594 completed questionnaires, yielding an effective response rate of 99%. The demographic characteristic questionnaire encompassed various factors, including region, sex, age, marital status, educational attainment, occupation, place of residence, family income, payment method, family history of CHD, smoking and drinking habits, understanding of CHD, frequency of medical visits, time of diagnosis (years), treatment experience, and Internet information search.

### 3.1 Sociodemographic data and patient characteristics

Sociodemographic data and patient characteristics are shown in Table 1. The majority of the patients were male (57.4%), married (77.4%), and farmers (50.8%). Additionally, most patients had an educational level equivalent to that of primary school (40.1%), resided in rural areas (53.7%), and reported an annual income between 1,000 and 1999 (33.8%). Furthermore, 56.4% of the patients had no family history of the disease. With respect to lifestyle habits, 55.9% were non-smokers and 49.7% were non-drinkers. Notably, 294 (49.5%) patients had been hospitalized 2–3 times, and 48.3% had prior experience with Internet information searches.

**Table 1:** Sociodemographic data and patient characteristics (*n* = 594).

Variable	Description	*N* = 594	Percentage (%)
Region	Nanyang	498	83.8
	Ningbo	96	16.2
Sex	Male	341	57.4
	Female	253	42.6
Age	45 to 59	203	34.2
	60 to 74	264	44.4
	≥75	127	21.4
Marital status	Married	460	77.4
	Other^a^	134	22.6
Educational attainment	Primary school and below	238	40.1
	Junior middle school	199	33.5
	Senior middle school	114	19.2
	Junior college and above	43	7.2
Occupation	Farming	302	50.8
	Labor	126	21.2
	Business	53	8.9
	Teaching	44	7.4
	Civil servant (government agencies)	34	5.7
	Other	35	5.9
Place of residence	City	275	46.3
	Rural	319	53.7
Income (¥)	<1,000	125	21.0
	1,000–1999	201	33.8
	2000–4,999	189	31.8
	>5,000	79	13.3
Payment method	NRCMS	300	50.5
	Medical insurance	229	38.6
	Uninsured	65	10.9
Family history of CHD	Yes	259	43.6
	No	335	56.4
Smoking habits	No	332	55.9
	Yes	202	34
	Quit smoking	60	10.1
Drinking habits	No	295	49.7
	Yes	225	37.9
	Quit drinking	74	12.5
Understanding of CHD	Not at all	133	22.4
	A little understanding	304	51.2
	More understanding	115	19.4
	Totally understood	42	7.1
Frequency of medical visits (No.)	1	213	35.9
	2–3	294	49.5
	>3	87	14.6
Time of diagnosis (years)	<1	166	27.9
	1–5	260	43.8
	5–10	130	21.9
	>10	38	6.4
Treatment experience	NO	179	30.1
	CAG	182	30.6
	PCI	212	35.7
	CABG	21	3.5
Internet information search	NO	307	51.7
	YES	287	48.3

a Single, divorced, or widowed.

### 3.2 The scores and grades of eHL, medical compliance behavior, and QOL

The scores and grades of eHL, medical compliance behavior, and QOL are shown in Table 2. The eHL scores of middle-aged and older adult patients with CHD are reported as 21 (13, 29). Among the participants, 224 individuals demonstrated good eHL, accounting for 37.7% of the total sample. The majority of patients exhibited a poor ability to utilize network information, evaluate information critically, and apply this information effectively. The medication compliance score was 5.5 (3.75, 6.75). Among the patients analysed, 376 (63.3%) were classified as having low compliance, 157 (26.4%) had moderate compliance, and only 61 (10.3%) had high compliance. The overall score for non-pharmacological adherence was 8 (7, 10) points. Within this category, a significant portion of 318 patients (53.5%) were identified with low compliance, followed by moderate compliance in 239 patients (40.2%) and high compliance in 37 patients (6.2%). Finally, the SAQ QOL score averaged 47.73 (36.36, 61.36) points. Notably, there were instances where QOL ratings varied significantly: 44 patients (7.4%) reported very poor QOL, 272 patients (45.8%) reported fair QOL, 233 patients (39.2%) reported good QOL, and 45 patients (7.6%) reported excellent QOL. Among middle-aged and older patients with CHD, the eHL scores were relatively low, their medical advice adherence was suboptimal, and their overall QOL was moderate.

**Table 2:** The scores and grades of eHL, medical compliance behavior, and QOL (*N* = 594).

Variable	Median (IQR)	Grade	*N* = 594	Percentage (%)
eHL	21 (13, 29)	Qualified	224	37.7
		Unqualified	370	62.3
Morisky	5.5 (3.75, 6.75)	Low adherence	376	63.3
		Moderate adherence	157	26.4
		High adherence	61	10.3
Non-medicine adherence	8 (7, 10)	Low adherence	318	53.5
		Moderate adherence	239	40.2
		High adherence	37	6.2
SAQ	47.73 (36.36, 61.36)	Very poor to poor	44	7.4
		Fail	272	45.8
		Good	233	39.2
		Excellent	45	7.6

eHL, eHealth literacy; SAQ, Seattle Angina Questionnaire; Results of the Mann–Whitney U test or the Kruskal–Wallis H test; IQR, Interquartile Range.

### 3.3 Relationships between eHL, medical compliance behavior, and QOL

The Spearman correlation coefficients for the relationships among eHL, compliance behavior, and QOL are shown in Tables 3, 4. A significant positive correlation was observed between eHL status and medication compliance (*r* = 0.397, *p* < 0.001). A significant positive correlation was observed between eHL status and non-medicine adherence compliance (*r* = 0.337, *p* < 0.001). Furthermore, eHL demonstrated a significant positive correlation with QOL (*r* = 0.539, *p* < 0.001).

**Table 3:** Relationships between eHL, medical compliance behavior, and QOL (*N* = 594).

Variable	eHL	Application ability	Evaluation ability	Decision-making ability
Medication adherence	0.397^**^	0.398^**^	0.375^**^	0.360^**^
Non-medicine adherence	0.337^**^	0.335^**^	0.325^**^	0.312^**^
QOL	0.539^**^	0.545^**^	0.497^**^	0.505^**^

eHL, eHealth literacy; QOL, quality of life. **p < 0.001. Results of Spearman’s correlation.

**Table 4:** Relationships between eHL, medical compliance behavior, and QOL (*N* = 594).

Variables	eHL	Application ability	Evaluation ability	Decision-making ability	Medication adherence	Non-medicine adherence	QOL
eHL	1	0.990^**^	0.964^**^	0.941^**^	0.397^**^	0.337^**^	0.539^**^
Application ability	0.990^**^	1	0.926^**^	0.900^**^	0.398^**^	0.335^**^	0.545^**^
Evaluation ability	0.964^**^	0.926^**^	1	0.931^**^	0.375^**^	0.325^**^	0.497^**^
Decision-making ability	0.941^**^	0.900^**^	0.931^**^	1	0.360^**^	0.312^**^	0.505^**^
Medication adherence	0.397^**^	0.398^**^	0.375^**^	0.360^**^	1	0.314^**^	0.423^**^
Non-medicine adherence	0.337^**^	0.335^**^	0.325^**^	0.312^**^	0.314^**^	1	0.357^**^
QOL	0.539^**^	0.545^**^	0.497^**^	0.505^**^	0.423^**^	0.357^**^	1

eHL, eHealth literacy; QOL, quality of life. ** *p* < 0.001. Resulted from Spearman correlation.

### 3.4 Predictors of eHL, medical compliance behavior, and QOL

Multivariable regression analysis identified several demographic and clinical characteristics associated with eHL, medication adherence, non-medication adherence, and QOL (Table 5). Approximately half of the variance in eHL was explained by this model (*R*^2^ = 50.7%). Compared with patients aged < 60 years, both the 60-74-year group (aOR = 0.518, 95% CI: 0.315–0.851, *p* = 0.009) and those aged ≥ 75 years (aOR = 0.133, 95% CI: 0.057–0.312, *p* < 0.001) had significantly lower eHL. Higher educational attainment was positively associated with eHL, with middle-school education (aOR = 1.717) and high-school/vocational education (aOR = 2.487) showing higher odds of adequate eHL compared with primary education or below. Patients with a history of cardiac intervention also demonstrated higher eHL, particularly those who underwent PCI (aOR = 3.461, *p* < 0.001) or CABG (aOR = 4.997, *p* = 0.012).

**Table 5:** Multiple regression analysis summary for the variables of eHL, medical compliance behavior, and QOL (*N* = 594).

Variable	*B*	SE	*p*	aOR	95% CI lower	95% CI upper
eHL						
60–74	0.658	0.254	0.009	0.518	0.315	0.851
≥75	2.019	0.435	0.000	0.133	0.057	0.312
Middle	0.540	0.302	0.073	1.717	0.950	3.101
High/Voc	0.911	0.345	0.008	2.487	1.263	4.894
CAG	0.550	0.311	0.077	1.734	0.942	3.190
PCI	1.242	0.301	< 0.001	3.461	1.919	6.243
CABG	1.609	0.643	0.012	4.997	1.418	17.611
Medication Adherence						
60–74	0.713	0.184	< 0.001	0.490	0.341	0.704
≥75	1.461	0.240	< 0.001	0.232	0.145	0.371
Smoker	0.435	0.179	0.015	0.647	0.456	0.920
Ex-smoker	0.544	0.268	0.042	0.580	0.343	0.982
CAG	0.254	0.215	0.238	0.776	0.509	1.183
PCI	0.101	0.221	0.648	1.106	0.717	1.707
CABG	0.481	0.468	0.304	1.618	0.647	4.050
Moderate	0.814	0.307	0.008	2.256	1.235	4.122
High	1.237	0.407	0.002	3.447	1.552	7.657
1,000–2000	0.274	0.233	0.239	1.316	0.833	2.077
2000–5,000	0.319	0.260	0.219	1.376	0.827	2.290
>5,000	0.536	0.313	0.087	1.709	0.925	3.157
Low/Moderate	0.682	0.265	0.010	0.506	0.301	0.850
Moderate/High	0.710	0.058	< 0.001	2.035	1.816	2.280
Non-Medication Adherence						
Male	0.532	0.181	0.003	0.588	0.412	0.837
Middle	0.067	0.204	0.740	1.070	0.718	1.595
High/Voc	1.280	0.260	< 0.001	3.596	2.162	5.981
Single	0.415	0.518	0.423	0.661	0.240	1.822
Divorced	0.050	0.330	0.880	1.051	0.550	2.008
Widowed	0.565	0.265	0.033	0.568	0.338	0.954
Smoker	1.166	0.206	< 0.001	0.312	0.208	0.467
Ex-smoker	0.097	0.325	0.766	1.102	0.583	2.082
Drinker	1.397	0.197	< 0.001	0.247	0.168	0.364
Ex-drinker	0.286	0.301	0.342	0.751	0.417	1.355
Moderate	0.966	0.280	0.001	2.627	1.519	4.544
High	1.338	0.410	0.001	3.810	1.705	8.510
Low/Moderate	1.411	0.231	< 0.001	0.244	0.155	0.383
Moderate/High	0.833	0.061	< 0.001	2.300	2.041	2.591
SAQ						
60–74	0.588	0.199	0.003	0.555	0.376	0.820
≥75	0.815	0.275	0.003	0.443	0.258	0.759
Single	0.479	0.577	0.406	0.620	0.200	1.919
Divorced	0.483	0.339	0.155	0.617	0.317	1.200
Widowed	0.274	0.267	0.305	0.761	0.451	1.283
1,000–1999	0.218	0.242	0.369	1.243	0.774	1.998
2000–4,999	0.791	0.271	0.004	2.205	1.296	3.752
>5,000	0.846	0.360	0.019	2.329	1.151	4.716
eHL_score	0.064	0.013	0.000	1.066	1.039	1.093
MMAS-8_score	0.176	0.057	0.002	1.193	1.067	1.333
Nonmed_score	0.135	0.043	0.002	1.145	1.053	1.245
Low/Moderate	2.116	0.459	0.000	8.295	3.371	20.409
Moderate/High	0.682	0.066	0.000	1.979	1.740	2.250

CAG, Coronary Angiography; PCI, Percutaneous Coronary Intervention; CABG, Coronary Artery Bypass Grafting; eHL, Electronic Health Literacy; SAQ, Seattle Angina Questionnaire; MMAS-8, The Morisky Medication Adherence Scale-8.

High/Voc = High school or vocational education level (reference group: primary education or below).

Moderate/High = Moderate-to-high-level group (reference: low level).

Low/Moderate = Low-to-moderate-level group (reference: high level).

aOR > 1 indicates a higher likelihood of better eHealth literacy, medication adherence, non-medication adherence, or quality of life compared with the reference group.

For example, “High/Voc” under eHL means that patients with higher education were more likely to have better eHealth literacy than those with a primary school education or below. aOR < 1 indicates a lower likelihood relative to the reference category (e.g., older age and smoking were associated with lower adherence and QOL).

For medication adherence (*R*^2^ = 20.7%), age again emerged as an independent predictor, with lower adherence observed in the 60-74-year (aOR = 0.490, *p* < 0.001) and ≥ 75-year (aOR = 0.232, *p* < 0.001) groups. Current smoking was associated with poor medication adherence (aOR = 0.647, *p* = 0.015), whereas patients with a higher understanding of CHD had better adherence (aOR = 3.447, *p* = 0.002). No significant associations were observed among prior revascularization procedures in this model. Regarding non-medication adherence (*R*^2^ = 24.2%), male patients (aOR = 0.588, *p* = 0.003), widowed individuals (aOR = 0.568, *p* = 0.033), current smokers (aOR = 0.312, *p* < 0.001), and drinkers (aOR = 0.247, *p* < 0.001) were less likely to adhere to lifestyle recommendations. In contrast, higher educational attainment (aOR = 3.596, *p* < 0.001) and greater CHD knowledge (aOR = 3.810, *p* = 0.001) were associated with better non-medication adherence.

The QOL model explained 49.9% of the total variance. Older age was associated with poorer QOL (60–74 years: aOR = 0.555, *p* = 0.003; ≥ 75 years: aOR = 0.443, *p* = 0.003), whereas higher income (>5,000/month: aOR = 2.329, *p* = 0.019) predicted better QOL. Living in Ningbo was independently associated with higher QOL (*β* = 0.603). Notably, higher eHL (aOR = 1.066, p < 0.001), better medication adherence (aOR = 1.193, *p* = 0.002), and greater adherence to non-pharmacological recommendations (aOR = 1.145, *p* = 0.002) were significant contributors to improved QOL. These findings highlight that older age, lower education level, and unhealthy lifestyle behaviors remain key barriers to digital health competence and adherence behaviors in patients with CHD. Conversely, patient knowledge, prior cardiac treatment experience, and higher socioeconomic status positively contributed to health literacy, self-management, and overall quality of life.

## 4 Discussion

This study examined eHL, compliance behavior (medication and non-medication adherence), and health-related QOL among middle-aged and older adults with CHD. Three key observations were made in this study. First, eHL, compliance behavior, and QOL were positively associated. Second, eHL varied systematically across sociodemographic and care-related characteristics. Third, both medication and non-medication adherence were linked to QOL in the expected directions. Taken together, these findings support the view that eHL and adherence behaviors are interrelated determinants of patient-centred outcomes in CHD.

Consistent with previous reports (29, 43), a substantial proportion of patients did not reach an adequate level of eHL. Lower eHL was observed among older adults and those with lower educational attainment or less frequent Internet use. These patterns accord with evidence that limited digital skills and reduced access to trustworthy online resources impede information acquisition and patient-provider communication, thereby weakening engagement with modern treatment and home-based care (25, 44). Conversely, higher education likely confers advantages in information seeking, appraisal, and application, which has been previously documented (45). From a clinical perspective, these data underscore the need to identify patients with low eHL and provide structured guidance for reliable online resources, together with training in information appraisal, to strengthen self-management in CHD (46).

Non-adherence to prescribed therapy and recommended lifestyle remains common worldwide (47). In our cohort, medication adherence was suboptimal for a considerable proportion of patients, in line with Padilha et al. (19) but lower than that reported by Nouamou et al. (48), likely reflecting differences in socioeconomic context and disease severity across samples. Factors associated with poorer adherence included older age and smoking, whereas higher family income, previous treatment experience, and better understanding of CHD were linked to better adherence. These associations are biologically and behaviorally plausible: ageing may increase regimen complexity and cognitive burden; tobacco use often co-occurs with lower risk perception; and economic constraints can limit access to medications. Disease knowledge may improve risk appraisal and self-efficacy, thereby facilitating therapy persistence.

For secondary prevention behaviors, education and CHD understanding were positively related to adherence, whereas smoking and alcohol consumption were inversely related patterns that mirror prior observations and highlight the role of health literacy and entrenched lifestyle habits. Marriage appeared protective, consistent with the supportive effects of spousal encouragement and shared routines on health-seeking behavior. These findings suggest that strengthening disease-specific knowledge and mobilizing family support could significantly improve lifestyle-related adherence in this population.

QOL in this cohort was at a moderate level and varied by age, marital status, residence, and income, with urban residence and higher income associated with better QOL, echoing Zhou et al. (49). Notably, both eHL and adherence behaviors were positively related to QOL, in agreement with prior evidence (50). This pattern aligns with the pathway proposed by Lin et al. (23): eHL may influence QOL both directly and indirectly by facilitating symptom understanding, reducing psychological distress and sleep problems, and promoting adherence to therapeutic and lifestyle recommendations (51). In other words, eHL appears to be a modifiable upstream factor that enables healthier behaviors and, ultimately, better patient-reported outcomes (44, 46). Evidence from e-health interventions further indicates that targeted digital strategies can enhance secondary prevention adherence and improve QOL in CHD (52, 53).

These results support the routine assessment of eHL in cardiac clinics, followed by stratified education using plain language, step-by-step demonstrations, and family involvement for patients with low eHL. Hospitals and public health agencies should curate accessible and trustworthy digital resources and provide brief training in online information appraisal. For patients at risk of non-adherence (older age, smokers, low income), combining medication counseling with behavioral support and social support mobilization may be particularly effective.

## 5 Strengths and limitations

The weakness of this study is that it is cross-sectional; it only shows associations between the outcome and explanatory variables, but not causal associations. Thus, varying outcomes may emerge in different temporal contexts. Furthermore, the relatively short duration of this study precludes an assessment of the long-term trajectory of the effects of eHL on medical compliance behavior and QOL. Given that data from this cross-sectional study were collected at a single point in time, establishing causal relationships between variables remains challenging.

Despite these limitations, our research is not without its strengths. For the first time, we specifically targeted middle-aged and older adult populations, used a validated questionnaire for data collection, and concurrently examined multiple variables to thoroughly elucidate the characteristics of this population. This study highlights the role of eHL in improving compliance behaviors and QOL among middle-aged and older adults with CHD. It provides evidence on how eHL influences these factors, benefiting doctor-patient interactions, encouraging patient engagement in care, and enhancing health outcomes. The results of this study suggest that healthcare providers can enhance compliance behavior and promote health by offering e-healthcare services tailored to individuals’ e-health literacy levels. Additionally, the positive correlation between e-health literacy, compliance behavior, and quality of life underscores its significance in healthcare delivery.

## 6 Conclusion

The findings of this study indicate a positive correlation between eHL, compliance behavior, and QOL in middle-aged and older adults with CHD. Thus, individualized interventions targeting eHL should be prioritized to improve patients’ QOL and prognosis. Improving eHL helps combat misinformation related to CHD on electronic platforms, enhances self-management and treatment adherence, and ultimately improves overall well-being. Healthcare professionals play a crucial role in supporting patients with low eHL, particularly by effectively communicating, guiding treatment adherence, and providing access to reliable digital health information. Nurses, in particular, should offer personalized health guidance and promote accurate online information-seeking behavior.

From an academic perspective, this study contributes to the growing body of research on electronic health literacy by integrating eHL, adherence, and QOL into a unified analytical framework among middle-aged and older patients with CHD. It provides novel evidence from the Chinese healthcare context, enriching the current understanding of how eHL influences health behavior and outcomes under different cultural and healthcare delivery conditions. Future research should employ longitudinal and intervention designs to establish causal relationships between eHL and health outcomes and to evaluate the effectiveness of tailored eHL-enhancing programmes across diverse populations and healthcare settings.

## Data Availability

The original contributions presented in the study are included in the article/supplementary material, further inquiries can be directed to the corresponding author.
