# Correction: Correlation analysis of electronic health literacy, compliance behavior, and quality of life in middle-aged and older patients with coronary heart disease: a cross-sectional study

**DOI:** 10.3389/fpubh.2026.1900467

**Published:** 2026-06-10

**Authors:** Suihua Sun, Ruru Guo, Yinan Wang, Leilei Jiao, Chenchen Zhao, Shuxin Qiao, Jingjing Wang

**Affiliations:** 1 Department for Teaching and Research in Community Nursing, School of Nursing and Health, Zhengzhou University, Zhengzhou, China; 2 Catheterization Laboratory, Department of Cardiology, Nanyang Central Hospital, Nanyang, China; 3 Department of Radiation and Medical Oncology Ward, The First Affiliated Hospital of Ningbo University, Ningbo, China

**Keywords:** CHD, EHL, middle-aged and older adults, compliance behavior, QOL

In the **Acknowledgements**, the mandatory copyright notice for the MMAS-8 scale was unintentionally omitted in the originally published manuscript.

The corrected **Acknowledgements** section appears below:

“Use of the ©MMAS-8 is protected by US copyright and trademark laws. Permission for use of the scale and its coding is required. A license agreement is available from MMAR, LLC, www.moriskyscale.com.”

The original version of this article has been updated.

